# Overexpression of *Arabidopsis* Molybdenum Cofactor Sulfurase Gene Confers Drought Tolerance in Maize (*Zea mays* L.)

**DOI:** 10.1371/journal.pone.0052126

**Published:** 2013-01-10

**Authors:** Yao Lu, Yajun Li, Jiachang Zhang, Yitao Xiao, Yuesen Yue, Liusheng Duan, Mingcai Zhang, Zhaohu Li

**Affiliations:** State Key Laboratory of Plant Physiology and Biochemistry, College of Agronomy and Biotechnology, China Agricultural University, Beijing, People's Republic of China; RIKEN Plant Science Center, Japan

## Abstract

Abscisic acid (ABA) is a key component of the signaling system that integrates plant adaptive responses to abiotic stress. Overexpression of *Arabidopsis* molybdenum cofactor sulfurase gene (*LOS5*) in maize markedly enhanced the expression of *ZmAO* and aldehyde oxidase (AO) activity, leading to ABA accumulation and increased drought tolerance. Transgenic maize (*Zea mays* L.) exhibited the expected reductions in stomatal aperture, which led to decreased water loss and maintenance of higher relative water content (RWC) and leaf water potential. Also, transgenic maize subjected to drought treatment exhibited lower leaf wilting, electrolyte leakage, malondialdehyde (MDA) and H_2_O_2_ content, and higher activities of antioxidative enzymes and proline content compared to wild-type (WT) maize. Moreover, overexpression of *LOS5* enhanced the expression of stress-regulated genes such as *Rad 17*, *NCED1*, *CAT1*, and *ZmP5CS1* under drought stress conditions, and increased root system development and biomass yield after re-watering. The increased drought tolerance in transgenic plants was associated with ABA accumulation via activated AO and expression of stress-related gene via ABA induction, which sequentially induced a set of favorable stress-related physiological and biochemical responses.

## Introduction

Drought stress is one major environmental stress that adversely affects crop growth and productivity worldwide. Developing drought-tolerant crops would be the most promising and effective approach to improving agricultural productivity and water use efficiency against drought and water shortage. Drought tolerance in plant involves perception of stress signals and subsequent signal transduction, resulting in activation of various physiological and metabolic responses [1]. Up to date, hundreds of genes and their related signaling pathways have been identified as important for drought tolerance, and genetic engineering using some of these genes has increased plant drought tolerance [2], [3].

ABA is a key component of the signaling system that integrates the adaptive response of plants to abiotic stress including drought and salinity. It is involved in plant responses to regulation of growth and development, including shoot and root growth, and leaf transpiration [1]. ABA accumulation in plant cells occurs quickly as plants respond to drought stress, which promotes expression of ABA-inducible genes [4] and stomatal closure to reduce transpirational water loss [5]. To some extent, putative high ABA content induced stomatal closure, which is important for plant tolerance of water stress [6]. Accordingly, an important strategy for plant drought tolerance is regulation of stomatal movement by ABA actions.

ABA *de novo* biosynthesis occurs in leaves, stems, and roots of most plant species primarily in plastids, but the last two steps occur in the cytoplasm where xanthoxin is converted to ABA [7], [8]. The 9-*cis*-epoxycarotenoid dioxygenase (*NCED*) is a rate-limiting enzyme in ABA biosynthesis which catalyses the cleavage of 9-*cis*-violaxanthin and/or 9-*cis*-neoxanthin to produce xanthoxin in plastids [9], [10]. Xanthoxin is converted to abscisic aldehyde by dehydrogenase/reductase in the cytoplasm [11]. Abscisic aldehyde is oxidized to ABA by aldehyde oxidase (AO) [12]. AO needs the sulphurylated form of a molybdenum cofactor (MoCo) for its activity [13], and the *LOS5* gene encodes the MoCo sulfurase involved in regulation of ABA biosynthesis [14].

The aforementioned steps show the molecular mechanism of ABA biosynthesis, and genetic engineering using some of these genes has improved plant drought tolerance. For example, *AtZEP*-overexpressing transgenic *Arabidopsis* showed smaller stomatal aperture, enhanced *de novo* ABA biosynthesis, and higher tolerance of osmotic stress than WT *Arabidopsis* [15]. Overexpression of *NCED* may increase endogenous ABA levels, trigger stomatal closure, and lead to higher drought tolerance in transgenic *Arabidopsis* [10], tobacco [16], creeping bentgrass [17] and transgenic tomato [2], [18], [19].

*LOS5* is an important gene that regulates the last step of ABA biosynthesis and enhanced expression in *Arabidopsis* is induced by drought, salt, and ABA treatment [14]. Overexpression of *LOS5* in rice under field conditions resulted in more spikelet fertility and yield than for non-transgenic plants [3]. Our early work showed that overexpression of *LOS5* in tobacco improved drought tolerance via reducing water loss and increasing antioxidant systems [20]. Maize is grown on more than 30 million ha annually in China, especially in China's semi-arid and arid regions where water shortage limits irrigation. However, maize is especially sensitive to water stress because of its relatively sparse root system [21], and this sensitivity to water stress can lead to dramatic fluctuations in yield due to frequent drought and poor irrigation management, as often the case in China. With the functions of stress-inducible genes well recognized, genetic manipulation is an effective approach for the enhancement of stress tolerance in crops.

The goal of the current study was to evaluate the effect of overexpression of *Arabidopsis LOS5* in maize subjected to drought stress. The study also aimed to explore the difference in stress–resistance mechanism between transgenic *LOS5* and WT maize, including their physiological and morphological responses under drought stress. Also, regulatory networks influenced by *LOS5* gene expression in maize were monitored by quantifying the expression of known stress-related genes.

## Results

### Generation of transgenic maize lines overexpressing *LOS5*

**Figure 1:**
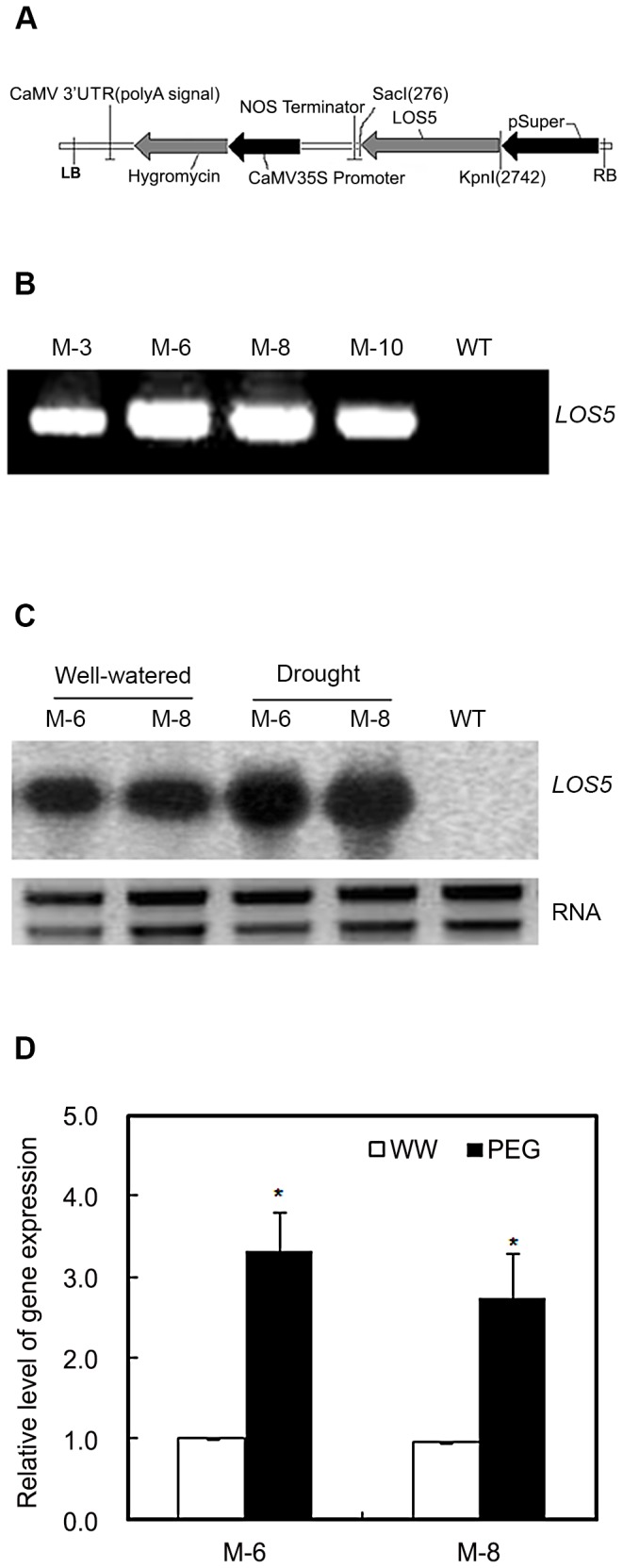
Schematic structure of the T-DNA and molecular analysis of *LOS5*-expressing maize. (A) T-DNA region of the vector pCAMBIA1300-*LOS5*. LB, left T-DNA border; RB, right T-DNA border; pSuper, ‘Super-Promoter’; Hygromycin, hygromycin phosphotransferase II gene; CaMV, cauliflower mosaic virus 35S promoter. (B) PCR analysis using two *LOS5*-specific primers to identify four independent T4 transgenic lines. (C) Northern analyses of T4 transgenic maize (lines M-6 and M-8) under well-watered and drought stress conditions. Moderate drought stress (D1) was applied to 21-d-old maize by maintaining 60% normal water supply for 5 d, whereas the control plants were watered normally. Total RNAs were isolated from uppermost fully expanded leaves of WT and transgenic maize and used for northern blotting. (D) Expression of *LOS5* under 20% PEG. Expression of *LOS5* was determined by RT-qPCR using RNAs isolated from 21-d-old WT and transgenic maize (lines M-6 and M-8) exposed to 20% PEG for 12 h. *Actin* gene was used as the control. Expression level of transgenic line was shown relative to the expression of transgenic line grown under well-watered condition. Error bars denote the standard deviation values, and asterisks indicated a significant difference (*P<0.05) compared with the corresponding controls.

The vector pCAMBIA1300-*LOS5* (Figure 1A) was introduced into maize via *Agrobacterium tumefacians*-mediated transformation. The primary transformed plants were designated as T0 plants, and seeds from self-fertilization of T0 plants were used to raise T1 progeny. A total of 16 independent maize transgenic lines were generated, and the transformations were confirmed by PCR analysis (Figure 1B). Two dominant lines, M-6 and M-8, were selected and homozygous T4 transgenic plants were used for drought-tolerance analysis.

Seedlings 21-d-old of WT and T4 transgenic maize had similar morphological characteristics under well-watered conditions (Table 1). The fresh weight of shoots and roots, number of visible leaves, and total leaf area exhibited similar values among WT and transgenic lines.

**Table 1:** Morphological characteristics of 21-d-old seedlings of WT and transgenic maize.

Lines	Fresh weight of shoot (g.plant^−1^)	Fresh weight of root (g.plant^−1^)	Number of leaves (No.plant^−1^)	Total leaf area (cm^2^)
WT	2.251±0.238	1.581±0.158	4	52.3±3.9
M-6	2.303±0.221	1.619±0.214	4	53.9±4.5
M-8	2.282±0.198	1.586±0.198	4	53.7±3.8

The data point are the mean of two independent biological experiments, and each experiment comprised five samples. Error bars denote the standard deviation values.

To investigate the role of overexpressing *LOS5* in transgenic maize under drought stress, RNA blot analysis was used to monitor *LOS5* transcription in leaves under different drought-stress conditions. Under well-watered conditions, the *LOS5* gene was transcribed in leaves of transgenic plants and not in WT plants (Figure 1C). Moreover, expression of *LOS5* of M-6 and M-8 maize was 3.3- and 2.8-fold greater than WT maize under drought stress (Figure 1D).

### *LOS5 o*verexpression improves ABA accumulation

**Figure 2:**
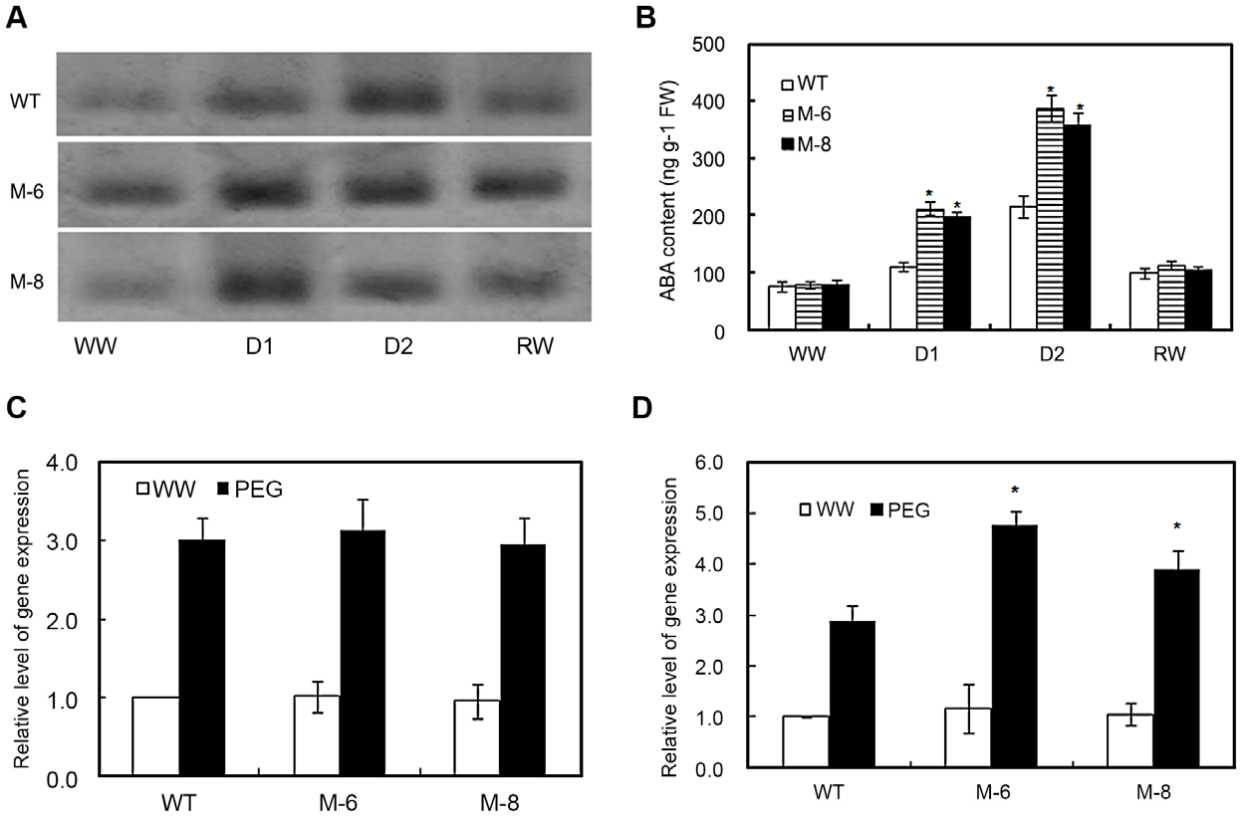
Aldehyde oxidase (AO) activity, ABA levels and expression of *ZmABA3* and *ZmAO1* in leaves. (A) Native PAGE assay for AO activity from transgenic maize (lines M-6 and M-8) and WT leaf extracts. WW, well-watered; D1, moderate drought stress (60% normal water supply) for 5 d; D2, severe drought stress (40% normal water supply) for 5 d; RW, re-watered for 2 d. (B) Changes in ABA content in transgenic maize exposed to drought stress. Drought stress treatment was same as (A). Expression of molybdenum cofactor sulphurase *ZmABA3* (C) and *ZmAO1* (D) under well-watered and 20% PEG conditions. The expression level of transgenic lines is shown relative to the expression of WT plants grown under well-watered condition. Error bars denote the standard deviation values, and asterisks indicated a significant difference (*P<0.05) compared with the corresponding controls.

The *LOS5* gene encoding MoCo sulfurase is involved in regulation of AO, which regulates the last step of ABA biosynthesis, so a native protein gel assay was used to analyze the AO activity of leaf extracts from transgenic (M-6 and M-8) and WT maize (Figure 2A). Under well-watered conditions, AO activity of M-6 and M-8 maize was 26 and 9% higher than that of WT maize (data no shown). Otherwise, the AO activities of transgenic lines M-6 and M-8 were 112 and 87% higher than those of WT maize under D1 treatment. However, there was no difference in AO activities between transgenic and WT plants under D2 treatment or after re-watering (except for M-6).

To determine whether overexpressing *LOS5* increased ABA levels in transgenic plants under drought stress, M-6 and M-8 maize under D1 treatment exhibited 78 and 90% higher ABA levels than WT maize (Figure 2B). Also, M-6 and M-8 lines under D2 treatment had 66 and 79% higher ABA content than WT maize. It was interesting to see that ABA concentrations were similar in transgenic and WT plants during normal growth conditions or after re-watering. Clearly overexpression of the *LOS5* gene in transgenic maize was strongly induced by drought stress.

Expression of molybdenum cofactor sulphurase gene (*ZmABA3*) in transgenic lines was the same or very similar to WT maize under well-watered conditions. Although drought markedly increased the expression of *ZmABA3* of transgenic and WT maize, similar levels were showed in expression of *ZmABA3* between transgenic and WT plants (Figure 2C). However, drought significantly enhanced the expression of *ZmAO1* of transgenic lines compared with WT maize (Figure 2D). There was no difference in expression of *ZmAO1* between transgenic and WT plants under well-watered conditions.

### *LOS5* overexpression decreases stomatal aperture to reduce water loss

**Figure 3:**
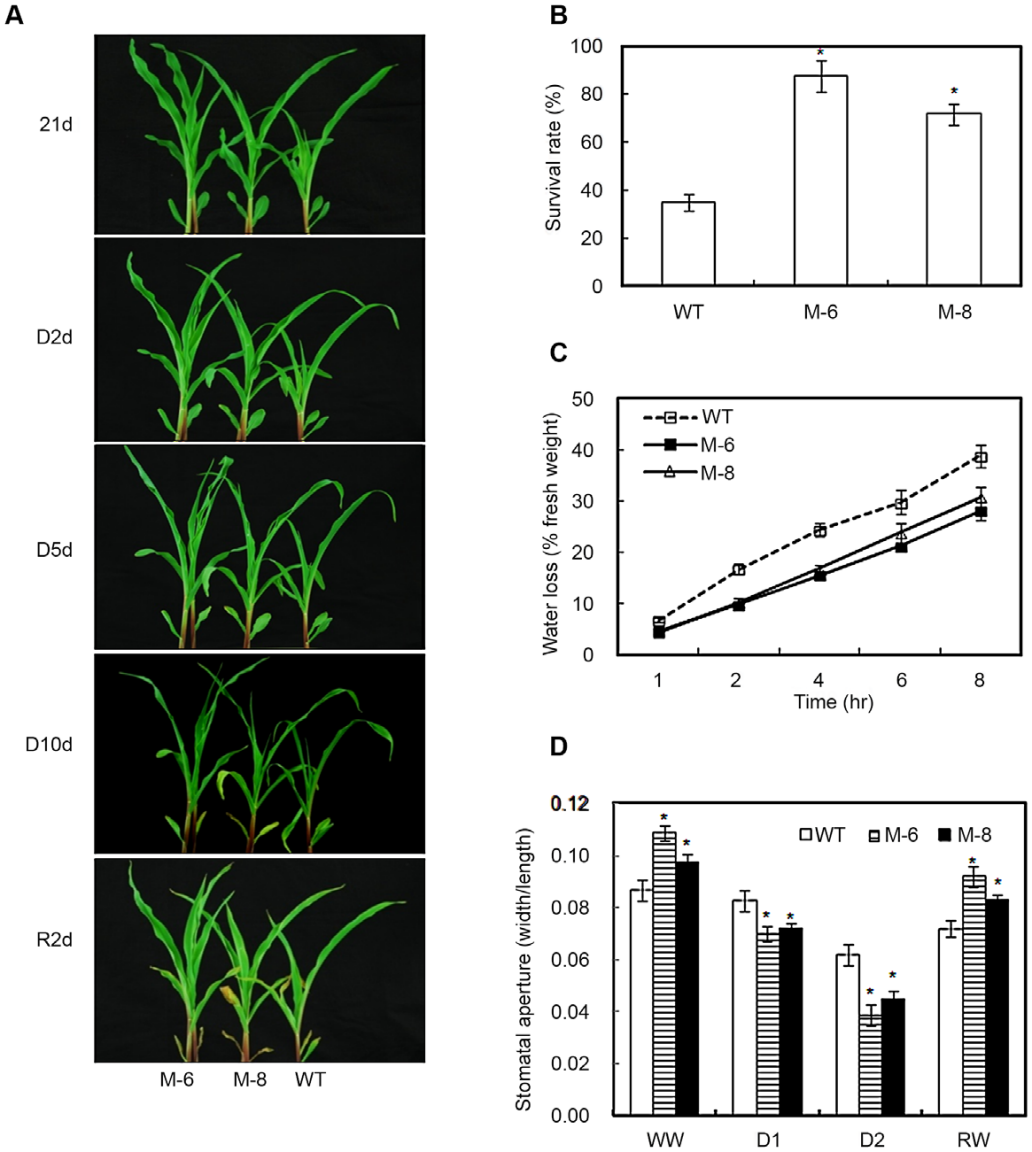
Drought-stress-tolerant phenotype, water loss and stomatal assay. (A) Drought-stress-tolerant phenotype of transgenic maize (lines M-6 and M-8). Drought stress treatment was applied to 21-d-old seedlings of WT and transgenic maize by completely withholding irrigation for 10 d, then re-watering for 2 d. 21 d, 21-d-old seedling; D 2 d, completely withholding irrigation for 2 d; D 5 d, completely withholding irrigation for 5 d; D 10 d, completely withholding irrigation for 10 d; R 2 d, re-watered for 2 d. (B) Survival rates of transgenic maize plants overexpressing *LOS5* under drought-stress conditions. Fifty 21-d-old seedling from each lines or WT were deprived of water for 14 d, watering was resumed for 7 d, and then the plants were scored for viability. Plants were considered dead if all the leaves were brown and there was no growth after 7 d of watering. Each column represents an average of two independent experiments with three replicates, and values represented the mean ±SD. (C) Comparison of transpirational water loss in detached leaves of WT and transgenic maize. Values represent the mean ±SD (n = 4). (D) Comparison of stomatal apertures of WT and transgenic maize under drought stress conditions. Drought stress treatment was imposed as described in Fig. 2 (A) above. Error bars denote the standard deviation values, and asterisks indicated a significant difference (*P<0.05) compared with the corresponding controls.

To investigate whether overexpression of *LOS5* improved water stress tolerance in maize, T_4_ transgenic seedlings were exposed to drought by withholding water. There were no marked differences in leaf turgor between WT and transgenic maize prior to drought stress (Figure 3A). After 2 d of withholding water, leaves of WT plants showed slight wilting, but transgenic lines M-6 and M-8 had normal turgid leaves. After 5 d of withholding water, leaves of WT plants were severely wilted and of transgenic lines M-6 and M-8 were moderately wilted. After 10 d of withholding water, leaves of M-6 maize showed less wilting than the M-8 line and of the WT maize completely wilted. After re-watering 2 d, leaves of the transgenic lines M-6 and M-8 showed less damage than those of WT maize. Survival rates were determined for WT and transgenic maize. Only 35% of WT maize recovered, whereas 72 to 88% of transgenic lines survived (Figure 3B).

Transpirational water loss of 21-d-old seedlings from lines M-6 and M-8 was 28 and 21% less than WT plants (Figure 3C). The reduced water loss by transgenic maize indicated that stomatal action was regulated by overexpressing *LOS5*. Under well-watered conditions, stomatal apertures of transgenic lines were larger than WT maize (Figure 3D). However, stomatal apertures under D1 condition of M-6 and M-8 lines were reduced by 15 and 13% compared with WT maize. Exposed to D2 treatment, stomatal apertures of M-6 and M-8 lines dropped 38 and 28% compared with WT maize. After D2 treatment, the plants were re-watered and recovery was evaluated after 2 d of normal water. Stomatal apertures of transgenic maize were larger than WT plants.

### *LOS5* overexpression holds high leaf water potential and RWC of transgenic maize under drought stress

**Figure 4:**
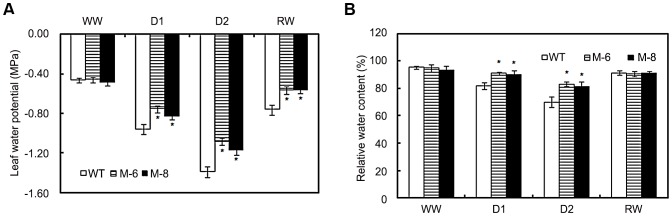
Leaf water status assay. Changes in leaf water potential (A) and relative water content (B) in WT and transgenic maize (lines M-6 and M-8) subjected to drought stress. Drought stress treatment was imposed as described in Fig. 2 (A) above. Error bars denote the standard deviation values, and asterisks indicate a significant difference (*P<0.05) compared with the corresponding controls.

To further characterize the drought response, the leaf water potential and RWC of transgenic and WT leaves under water stress conditions were evaluated. Under well-watered conditions, leaf water potential of transgenic lines was similar to WT maize (Figure 4A). Drought stress caused the leaf water potential of transgenic and WT plants to decline, but that of transgenic lines was much higher than WT maize. After re-watering, leaf water potential of transgenic and WT plants was restored, and values of transgenic lines were higher than those of WT maize.

The RWC under drought stress was maintained at a higher level in transgenic *LOS5* leaves than in WT maize (Figure 4B). For example, under D1 treatment, the RWC of M-6 and M-8 lines were 11 and 10% higher than that of WT maize and under D2 treatment it was 19 and 16% higher. The RWC was similar between transgenic and WT plants under well-watered condition or after re-watering.

### Low cell membrane damage of transgenic maize under drought stress

**Figure 5:**
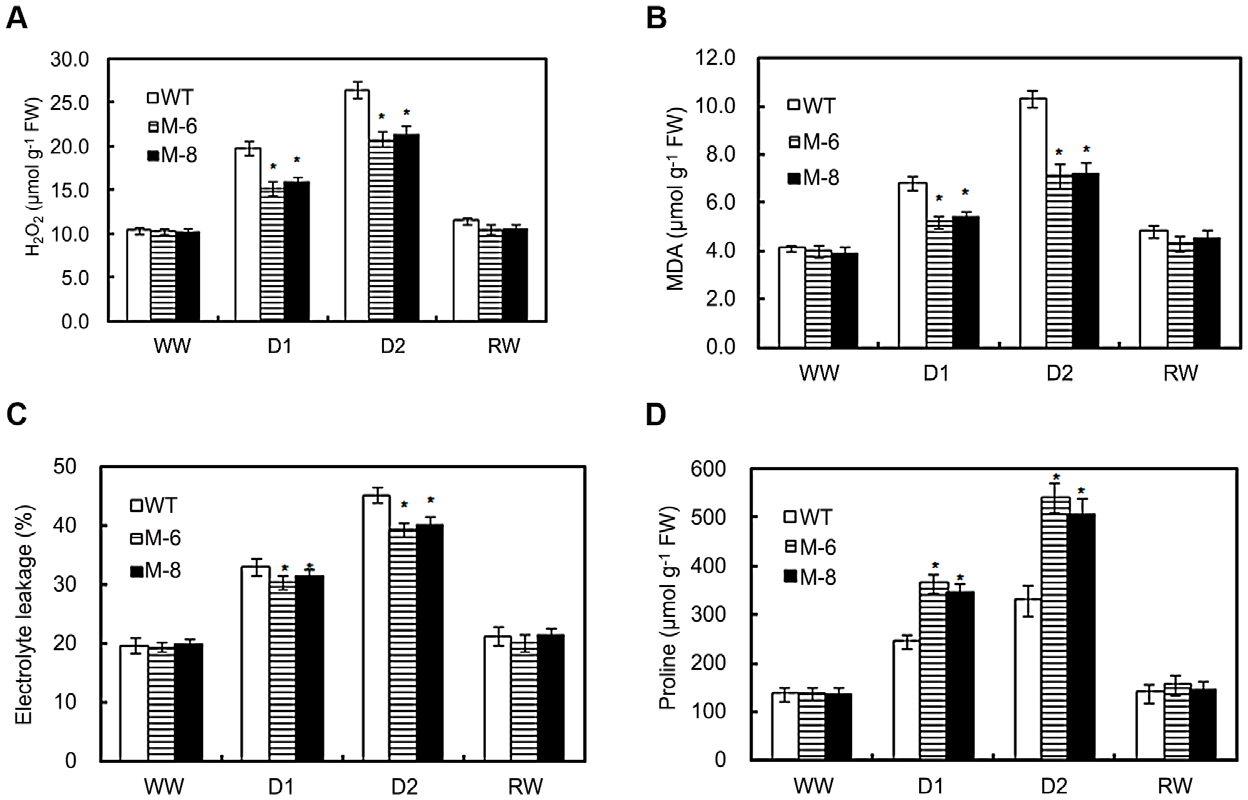
Cell membrane damage and non-enzymatic antioxidants assay. Changes in H_2_O_2_ content (A), MDA content (B), electrolyte leakage (C), and proline content (D) in WT and transgenic maize (lines M-6 and M-8) subjected to drought stress. Drought stress treatment was imposed as described in Fig. 2 (A) above. Error bars indicate SE, and asterisks indicate a significant difference (*P<0.05) compared with the corresponding controls.

Membrane damage to transgenic and WT maize under water deficit stress can be assessed by H_2_O_2_ accumulation, electrolyte leakage and MDA content. When exposed to D1 drought stress, M-6 and M-8 lines produced 30 and 25% less H_2_O_2_ than WT maize, and under D2 treatment produced 27 and 24% less H_2_O_2_ than WT maize (Figure 5A). However, for well-watered or re-watered plants, H_2_O_2_ contents were similar in transgenic and WT plants.

Electrolyte leakage and MDA content of transgenic and WT maize increased gradually with increasing water stress but was markedly lower in transgenic lines than WT plants under drought stress (Figure 5B, C). Electrolyte leakage and MDA content were similar between transgenic and WT maize for well-watered condition or after re-watering.

Proline accumulation is one positive response to drought that helps minimize dehydration in many plant species. WT and transgenic maize under well-watered condition had similar proline contents, but the contents increased with increasing water stress (Figure 5D). For example under D1 drought-stress, proline contents of M-6 and M-8 lines were 49 and 42% higher than those of WT plants and under D2 treatment were increased by 63 and 53% compared with WT maize. After re-watering, proline contents of transgenic and WT plants were similar and recovered to levels similar to well-watered plants that never were exposed to drought stress.

**Figure 6:**
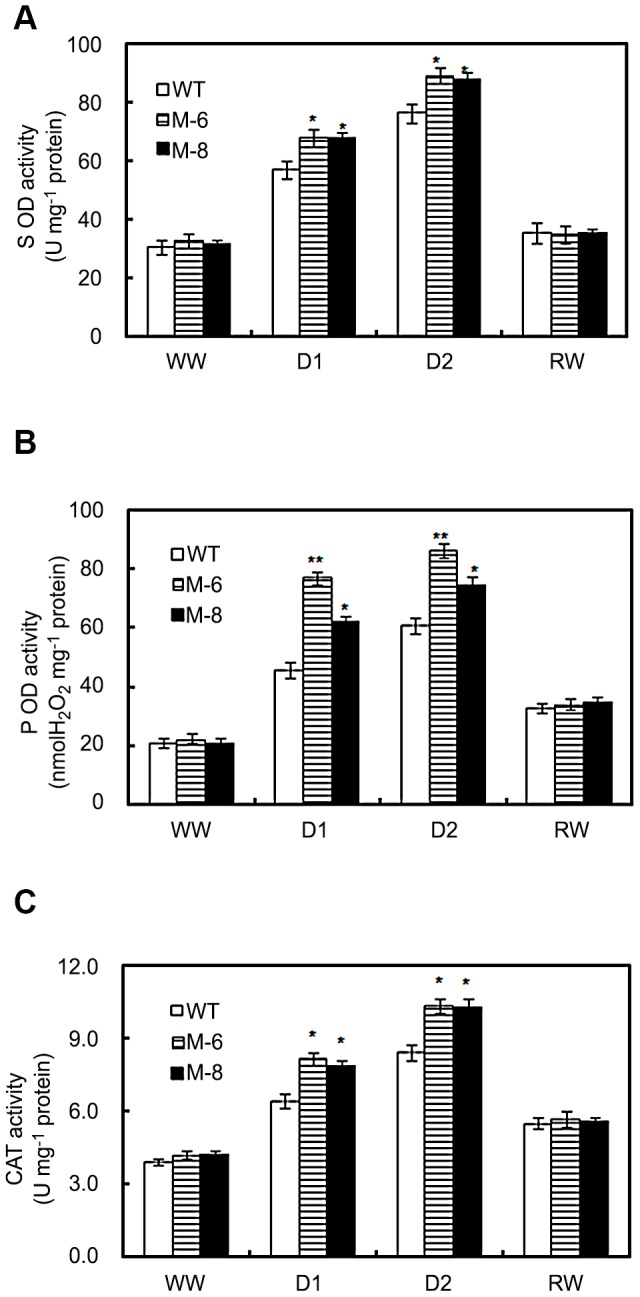
Antioxidant enzyme activities assay. Activities of SOD (A), POD (B), and CAT (C) in WT and transgenic maize (lines M-6 and M-8) subjected to drought stress. Drought stress treatment was imposed as described in Fig. 2 (A) above. Error bars denote the standard deviation values, and asterisks indicate a significant difference (*P<0.05; ** *P*<0.01) compared with the corresponding controls.

To assess whether *LOS5* overexpression affected activated oxygen production, which leads to damaged cell structures, enzymatic antioxidants were measured. Under well-watered conditions, activities of catalase (CAT), superoxide dismutase (SOD), and peroxidase (POD) were similar in transgenic and WT maize (Figure 6). When plants were drought stressed, the activities of CAT, POD and SOD increased greatly and were higher in transgenic maize than WT plants. After re-watering, the activities of CAT, POD and SOD in transgenic and WT plants decreased rapidly and were similar between transgenic and WT maize.

### *LOS5* overexpression enhances abiotic stress-related genes

**Figure 7:**
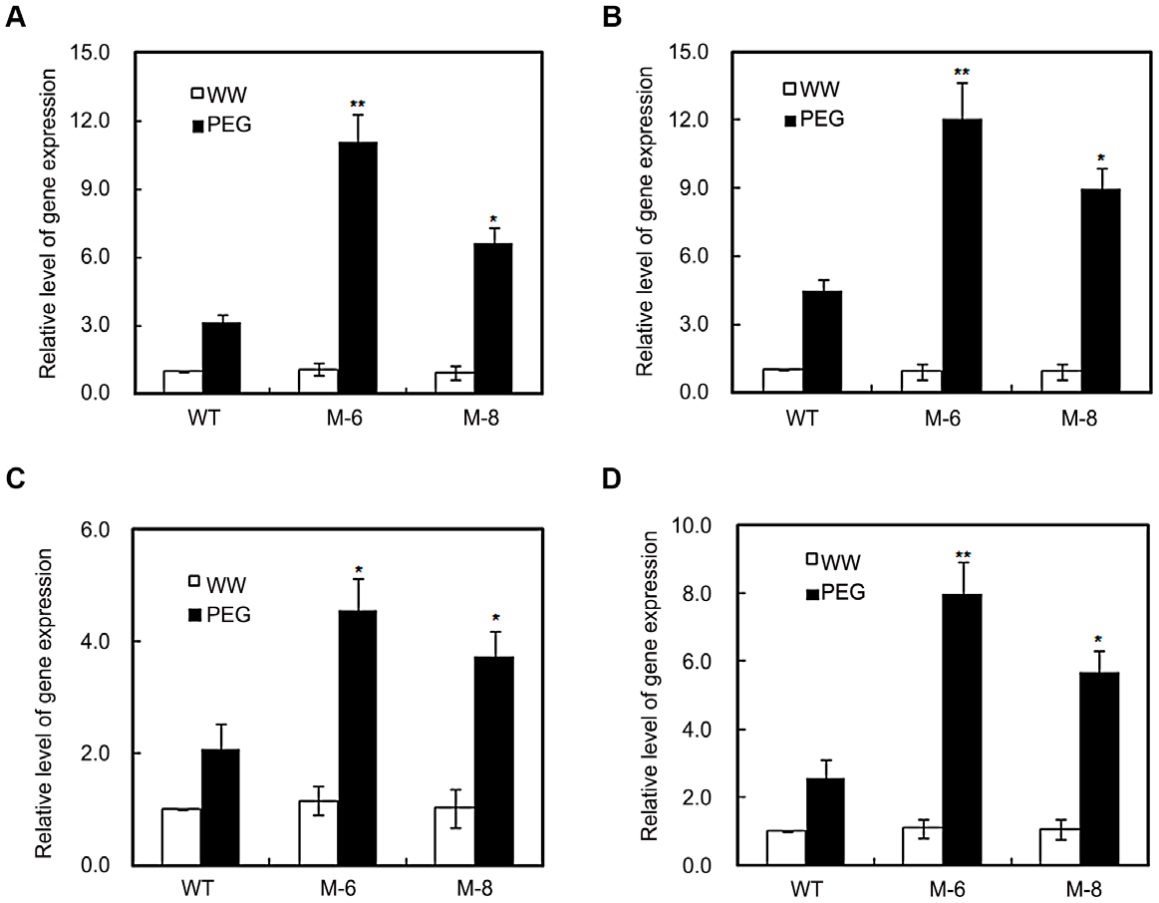
RT-qPCR analysis of stress-responsive genes. RNA levels of *Rad 17* (A), *NECD1* (B), *CAT1* (C), and *ZmP5CS1* (D) genes were determined by RT-qPCR using RNAs isolated from 21-d-old WT and transgenic maize (lines M-6 and M-8) exposed to 20% PEG. *Actin* gene was used as the control. The expression level of transgenic lines is shown relative to the expression of WT plants grown under well-watered condition. Error bars denote the standard deviation values, and asterisks indicate a significant difference (*P<0.05; ** *P*<0.01) compared with the corresponding controls.

We evaluated whether overexpression of *LOS5*, which increased drought tolerance in maize, lead to phenotypic changes in the expression patterns of stress-responsive genes in transgenic maize. In real-time PCR analysis under well-watered conditions, the expression of abiotic stress-related genes, such as *Rad17*, *NCED1*, *CAT1* and *ZmP5C1*, was similar for transgenic and WT maize. However, under drought stress, it was markedly higher in transgenic lines than WT maize (Figure 7). For example under drought stress, the expression of *Rad17* in M-6 and M-8 leaves was 3.5- and 2.1-fold greater than in WT maize (Figure 7A). Expression of *NCED1* (*vip14*) under drought stress in M-6 and M-8 lines was by 2.7- and 2.0-fold greater than for WT maize (Figure 7B). Expression of *CAT1* increased under drought stress in all plants but was much higher in transgenic lines than WT maize (Figure 7C). Expression of *ZmP5CS1* under drought stress in lines M-6 and M-8 was 3.1- and 2.2-fold higher than for WT maize (Figure 7D).

### Transgenic maize exhibited more robust root systems and biomass

Transgenic and WT maize were subjected to D2 treatment for 5 d and then were returned to normal water supply for 5 d. *LOS5* -expressing maize produced more dry mass than WT, and in both cases, the difference in root biomass was markedly greater than shoot biomass. For example, lines M-6 and M-8 produced 19 and 15% more dry shoot mass and 33 and 25% more dry root mass than WT maize (Table 2). After drought stress, the root/shoot ratio of M-6 and M-8 plants was 13 and 10% higher than that of WT maize. Thus, overexpression of *LOS5* in maize under water stress significantly improved their performance, which probably was primarily due to the increased root system of *LOS5*-expressing plants.

**Table 2:** Biomass assay in overexpression *LOS5* transgenic maize.

Lines	Shoot dry weight (g.plant^−1^)		Root dry weight (g.plant^−1^)		Root/Shoot	
	Well-watered	Drought	Well-watered	Drought	Well-watered	Drought
WT	1.05±0.07	0.59±0.04	0.42±0.06	0.24±0.02	0.40±0.02	0.40±0.02
M-6	1.07±0.14	0.70±0.02*	0.41±0.04	0.32±0.03*	0.38±0.03	0.45±0.01*
M-8	1.05±0.05	0.68±0.05*	0.42±0.07	0.30±0.01*	0.39±0.03	0.44±0.01*

Seedlings 21-d-old WT and transgenic maize (lines M-6 and M-8) were exposed to severe drought stress (D2) by withholding water for 5 d and then restoring it for 5 d. The data points are the mean of two independent biological experiments, and each experiment comprised ten samples. Error bars denote the standard deviation values, and asterisks indicate a significant difference (*P<0.05) compared with the corresponding controls.

## Discussion

A feasible strategy for increasing abiotic stress tolerance in plants is through applied plant biotechnology, which relies on expression of genes involved in signaling and regulatory pathways [22], or genes encoding proteins conferring stress tolerance [23], or enzymes present in pathways [24]. Genetic engineering is intensively explored to enhance plant stress tolerance, and several engineered plants have improved stress resistance phenotypes [3], [18]. To test whether this strategy could improve maize performance under drought conditions, transgenic maize that overexpressed the *Arabidopsis LOS5* gene was generated. When exposed to drought stress, the expression of *LOS5* was greatly upregulated in transgenic M-6 or M-8 maize although transcription of the transgene was driven by the constitutive super promoter (Figure 1C, D). This phenomenon was also observed in rice expressing *SaVHAC1* gene [25], transgenic *betA* gene in wheat [26], cotton [27] and maize [28]. Several studies have reported that transgene transcript of constitutive expression of a stress response gene in homologous or heterologous systems is induced by environmental stresses [10], [25], [28], [29]. The expression of genes at the cellular level is involved in transcriptional and posttranscriptional mechanisms, and posttranscriptional control of transcript accumulation is an important mechanism for gene regulation under stress [30], [31]. The results in this study suggested that a posttranscriptional regulation mechanism might be involved in controlling the stability of *LOS5* transcript. These results were in consistent with the observations that the ABA level in transgenic plants increased much more than that in WT plants after drought stress (Figure 2B).

Map-based cloning revealed that *LOS5* encoded a molybdenum cofactor sulphurase, and sulphurase catalyzed production of a sulfurylated molybdenum cofactor required by aldehyde oxidase (AO), which functions in the last step of ABA biosynthesis and functioned indirectly in ABA biosynthesis [14]. In our research with maize, overexpressing *LOS5* evidently increased the AO activity under two drought stress conditions (Figure 2A). Then, ABA accumulation was markedly enhanced in M-6 or M-8 transgenic maize subjected to drought stress (Figure 2B). These observations are supported by the model for stress induction of ABA biosynthesis [32], whereby an initial increase in ABA from overexpression of one ABA biosynthetic gene, such as *LOS5/ABA3*, could result in increased expression of other ABA biosynthetic genes, *AAO3* [33] and *LOS6/ABA1* [32]. Collectively, these genes would lead to a sustained increase in *de novo* ABA biosynthesis [18], [32].

We have shown that it was possible to reliably generate transgenic maize with high ABA content by manipulating a single key ABA biosynthetic gene under drought stress. Overexpressing *LOS5* did not enhance the expression of *ZmABA3* compared to WT maize under well-watered or drought conditions (Figure 2C), but markedly increased the expression of *ZmAO1* and AO activity which led to ABA accumulation in maize leaves under drought stress (Figure 2). However, under well-watered conditions, the expression of *ZmAO1* and ABA contents in *LOS5* transgenic maize were very similar to those of WT maize, which was inconsistent to overexpression of *NCED* in plants [2], [10], [19], [34]. *NCED* catalyzes the first specific step in ABA biosynthesis and affects ABA production when overexpressed or underexprssed [34], whereas the *LOS5* gene is stress-induced, and its transcript increased in response to drought, ABA, NaCl, and PEG treatments [14]. Otherwise, MoCo sulfurase (*LOS5*) convertion of di-oxygenated MoCo to mono-oxygenated MoCo is required to activate ABA aldehyde oxidase and indole-3-acetaldehyde oxidase, which are involved in ABA and IAA biosynthesis [35]. These observations suggested that constitutive expression of *LOS5* in maize could not promote ABA accumulation under well-watered or re-watering conditions, and might regulate indole-3-acetaldehyde oxidase involving IAA biosynthesis. The effect of *LOS5* overexpression on regulating auxin biosynthesis requires further investigation.

ABA is a key component of the signaling system that integrates the adaptive response of plants to drought and osmotic stress. Under drought stress, ABA accumulation was critical for stomatal closure that led to reduced transpirational water loss and induced expression of drought- and desiccation-tolerant genes [1]. Under drought stress, overexpression of *LOS5* in maize induced much higher ABA concentrations than non-transgenic plants. Transgenic lines had smaller stomatal apertures than WT maize, which led to less water loss in transgenic plants under drought conditions (Figure 3).

Overexpression of *LOS5* in maize showed markedly lower transpiration rates than WT plants, which led to reduce wilting in transgenic lines under drought conditions (Figure 3). Otherwise, overexpression of *LOS5* in maize leaves under drought stress resulted in higher RWC and lower leaf water potential than WT plants (Figure 4), which was an important strategy for transgenic plants to conserve water capability to reduce wilting. Our results were consistent with overexpression of *NCED* that led to increased ABA production and reduced leaf transpiration under drought conditions, which consequently increased the drought tolerance of transgenic plants [2], [10], [19].

Overexpression of *LOS5* under drought stress altered the expression of ABA-regulated genes (Figure 7). The maize ABA responsive gene *Rab17* is induced by water deficit, ABA, and desiccation in embryo and vegetative tissues [36]. Expression of *Rab 17* under drought stress in *LOS5-*overexpressing lines was 2.1- to 3.5-fold higher than in WT maize (Figure 7A). ABA is synthesized from C_40_-carotenoids, in which the oxidative cleavage of *cis*-epoxycarotenoids by *NCED* is the rate-limiting step of ABA biosynthesis in higher plants [37]. Expression of *NCED* is induced by drought and salt stress [16], [38]. Under drought stress, lines M-6 and M-8 overexpressing *LOS5* markedly enhanced the expression of *NCED1* compared to WT maize (Figure 7B). These results suggested that overexpressing *LOS5* promoted ABA accumulation thereby regulating expression of ABA responsive and biosynthetic genes.

Stress-induced production of reactive oxygen species (ROS) is a common metabolic response to environmental stress in plants [39]. ROS are signaling molecules that regulate plant-protective stress responses including ABA-induced activation of stomatal closure and induction of defense gene expression [40]. Water-stress-induced ABA accumulation regulated ABA-stress-responsive gene expression including ROS network genes such as SOD, APX and CAT [4], [41]. Overexpressing *LOS5* in maize greatly increased expression of *CAT1* compared to WT plants (Figure 7C), and the activity of CAT under drought stress in *LOS5* -expressing maize was higher than in WT plants (Figure 6C), which resulted in reduced H_2_O_2_ accumulation (Figure 5A) and cytoplasmic damage as detected by electrolyte leakage (Figure 5C).

*LOS5*-expressing maize under drought stress promoted accumulation of proline (Figure 5D), and the expression of *ZmP5SC1* was increased 2- to 3-fold compared to WT plants (Figure 7D). Stress-induced *P5CS1* (a key enzyme in proline biosynthesis) gene expression under osmotic stress required ABA [14], [42]. It is possible that *LOS5*-overexpressing plants under drought stress could accumulate proline by overproducing ABA. Our results clearly indicate that overexpression of *LOS5* enabled maize to detoxify ROS efficiently (Figure 5) and to enhance drought stress tolerance via mobilizing ROS-scavenging enzymes and activating signaling molecules that regulate ROS-scavenging genes. In this context, plants with a putative high ABA level might be most tolerant to stressful conditions [6].

In conclusion, overexpression of *LOS5* in maize subjected to drought stress increased drought tolerance by regulating AO activity to promote ABA accumulation. ABA accumulation in transgenic maize exposed to drought stress reduced water loss, and activated expression of stress-regulated genes that alleviated membrane damage. These data provide important insights into application of an ABA-related biosynthesis gene and significantly furthers our understanding of stress gene regulation and stress tolerance.

## Materials and Methods

### Construction of the binary vector and transformation

A constitutive super promoter, which consists of three copies of the octopine synthase enhancer in front of the manopine synthase promoter, was cloned as a *Sal*I–*Xba*I fragment into the pCAMBIA 1300 binary vector containing a hygromycin-resistant selectable marker (Figure 1A). *LOS5* cDNA of *Arabidopsis* was cloned as an *Xba*I–*Kpn*I fragment downstream of the super promoter in the modified pCAMBIA 1300 [29]. The recombinant plasmid was introduced into the *A. tumefaciens* strain *EHA105*, which was used to transform maize.

Transformation of maize inbred line Zheng 58 immature embryos was modified as described in Frame et al. [43]. Immature zygotic embryos (2 mm) were dissected and inoculated in *A. tumefaciens* suspension for 5 min. After infection, embryos were transferred to the surface of cocultivation medium and incubated in the dark at 20°C for 3 d and then transferred to resting medium at 25°C for 7 d. Infected embryos were transferred to selection medium 6 weeks later. Small pieces of Type II callus were regenerated on regeneration medium for 14 d. Mature somatic embryos were transferred to shoot induction medium or rooting medium to form plantlets with fully formed shoots and roots in the growth chamber at a light intensity of 50 µmol m^−2^ s^−1^. Transgenic maize with hygromycin resistance plants were transplanted into the pots (15×15×20 cm) filled with a mixture of vermiculite and sand (1∶1; v/v) and grown in the greenhouse.

### Polymerase chain reaction (PCR) analysis of transgenic plants

PCR analysis was carried out assaying T0 and T3 maize lines carrying the *LOS5* gene. Genomic DNA was isolated from expanding leaves of 21-d-old transgenic maize at V_2_ growth stage and untransformed WT plants by the cetyltrimethylammonium bromide method [44]. Equal amounts of 200 ng of total DNA were amplified in 50 µl reactions using specific primers for *LOS5* gene, forward primer 5′-CCTGATGGCTCTTGGTTTGGCTAC -3′ and reverse primer 5′-TTCCACTGACGACGGTTCCATTCC -3′ to amplify a 325 bp sequence from the *LOS5* gene coding region. The PCR reactions were conducted for an initial denaturation at 95°C for 5 min, followed by 35 cycles of 30 s at 94°C, 45 s at 55°C, and 30 s at 72°C, and a final extension at 72°C for 10 min. PCR products were separated by electrophoresis on a 1% (w/v) agarose gel.

### RNA isolation and RNA blot analysis

Total RNA was isolated from fresh leaves of 21-d-old T4 transgenic and WT maize, grown normally or maintaining 60% normal water supply for 5 d, with the TRIZOL reagent (Invitrogen GmbH, Karlsruhe, Germany) and RNAeasy columns (Qiagen, Hilden, Germany) according to the manufacturer's protocols. Leaves from the maize seedlings, 2 g per sample, were collected and ground into fine powder in liquid nitrogen, and then 100 mg of homogenized powder was added to 1 ml TRIZOL and incubated at 60°C for 5 min. Samples were centrifuged at 10 000 rpm for 10 min, and the supernatant was transferred to a new tube. Then 200 µl chloroform were added and incubated at room temperature for 2–3 min. Samples were again centrifuged as described above, and the aqueous supernatant was transferred to the Qia shredder column and centrifuged for 30 s at 10 000 rpm. A 350 µl aliquot of RLT buffer (plus b-mercaptoethanol) and 250 µl absolute ethanol were added to the flow-through and passed through an RNAeasy spin column. The quality of RNA was checked on a 1% agarose gel. RNA concentration was calculated using Nanodrop 2000 according to the manufacturer's instructions (Thermo Scientific, Wilmington, DE, USA), and then RNA samples were transferred onto nylon membranes. The RNAs were immoblized to the membrane at 1200 µJ/cm^2^ for 12 s, airdried and then baked for 2 h at 80°C in a vacuum oven. The membrane was prehybridized for 1 h at 64°C in the hybridization solution [1% BSA, 1 mM EDTA (pH 8.0), 0.5 mM Na_2_HPO_4_ (pH 7.2), 7% SDS]. Then hybridization at 64°C was performed overnight with the denatured ^32^P-labelled probe made by *LOS5* gene special primers (forward primer 5′-GGGAAAGGGTGGAGGAGT-3′ and reverse primer 5′- GTAGCCAAACCAAGAGCC-3′). The membrane was washed with solution I [0.5% BSA, 1 mM EDTA (pH 8.0), 40 mM Na_2_HPO_4_ (pH 7.2), 5% SDS] at 64°C for 5 min, and two times with solution II [0.1 mM EDTA (pH 8.0), 40 mM Na_2_HPO_4_ (pH7.2), 1% SDS] at 64°C for 10 min each. The membrane was wrapped in saran wrap and exposed to a phosphor screen for 2–5 h. Radioactivity was detected by scanning the phosphor screen using a phosphor imager [45].

### Real-time quantitative PCR (RT-qPCR) analysis

Seedlings 7-d-old of WT and T4 transgenic maize were placed in a box with nutrient solution and grown in a growth chamber. After solution culture for 14 d, plants were subjected to water deficit induced by 20% PEG in the nutrient solution, as had been selected in a preliminary experiment. After 12 h water deficit stress, expanding leaves of transgenic and WT were collected in liquid nitrogen before isolation of RNA. Total RNA was isolated using TRIZOL® reagent (Invitrogen, CA, USA) and purified using Qiagen RNeasy columns (Qiagen, Hilden, Germany) according to the instructions of the manufacturer. Reverse transcription was performed using Moloney murine leukemia virus (M-MLV; Invitrogen) according to the method described by Zhang et al. [46]. Primer Express program 3.0 (Applied Biosystems, Foster, CA, USA) was used to design the primers for the genes chosen: *LOS5*, forward 5′-TGATGCTGCAAAGGGTT GTGCTAC-3′ and reverse 5′-AATTGAAGCAGCAACAGTGCCTCC-3′; *ZmAO1*, forward 5′-GGGAGGCTGTGTACGTTGAT -3′ and reverse 5′-TCTCCACCGCTTGGAATATC-3′; *Zm ABA3*, forward 5′- CGGCAGGTGTACTTTGGGCAAA-3′ and reverse 5′-CGGGGTCCTGATTC GGTCACTCAG -3′; *Rab17*, forward 5′-CCCATAAGTACAGTGGCTGTGCT-3′ and reverse 5′-ACGTACAAATTCACCCCACAAGTA-3′; *NCED1 (Vp14)*, forward 5′-AGTTGTTGTCACCCAG TCCAG-3′ and reverse 5′-CACGCACCGATAGCCACA-3′; *ZmP5CS1*, forward 5′-ACTGCAA TGTCCACTTATCC-3′ and reverse 5′-TAACCTAGACTAGACACAGC-3′; *CAT1*, forward 5′-CTAACAGGCTGTCGTGAGAAGTG-3′ and reverse 5′-TGTCAGTGCGTCAACCCATC-3′; *β-actin*, forward 5′-GATTCCTGGGATTGCCGAT-3′ and reverse 5′-TCTGCTGCTGAAAAG TGCTGAG-3′, and the *Actin* gene was chosen as an internal control to normalize all data. Real-time quantitative RT-PCR was performed on a 7500 real-time PCR system (Applied Biosystems) using SYBR® Premix Ex Taq ™ (Perfect Real Time) (TaKaRa Code: DRR041A). According to the manufacturer's protocol, 1.5 µL cDNA, 0.4 µL PCR forward/reverse primer (10 µmol), 10 µL 2×SYBR® Premix Ex Taq™ and 0.4 µL ROX Reference Dye II (50×) were suspended in a final volume of 20 µL with ddH_2_O. RT-qPCR cycling conditions consisted of an initial polymerase activation step at 95°C for 30 sec, 40 cycles of 5 sec at 95°C, and 35 sec at 60°C. Melt-curve analysis was performed to monitor primer-dimer formation and amplification of gene specific products. The relative quantification method was used to evaluate quantitative variation between replicates.

### Plant material and growth conditions

Seeds of WT and T4 transgenic maize (lines M-6 and M-8) were planted into pots (15×15×20 cm deep) filled with a mixture of vermiculite and sand (1∶1; v/v) and grown in a growth chamber with a 14 h photoperiod at a 25/30°C night/day temperature cycle, 400 µmol m^−2^ s^−1^ irradiance (enhanced with high-pressure sodium lamps), and a relative humidity of 60%.

Drought stress was induced in 21-d-old seedlings of WT and T4 transgenic maize by completely withholding irrigation for 10 d. Drought-stress-tolerant phenotypes of transgenic lines were observed, and the number of wilted plants was scored and photographed. Then, the wilted plants were re-watered and resumed growth; drought-stress-tolerant phenotypes of transgenic maize after 2 d were recorded. Survival rate was recorded after 7 d of recovery from 14 d of drought stress and was defined as the number of healthy plants divided by the total number (50 plants) of each lines or WT.

Drought experiments also were conducted with WT and T4 transgenic maize grown in the pots as described above. Plants were watered to capacity daily by providing about 400 ml water per pot. After 21 d of growth with normal water supply, uniform plants were divided into four groups: well-watered group, moderate drought group (D1, 60% normal water supply), severe drought group (D2, 40% normal water supply), and re-watered group (re-watered after D2 treatments). For the drought treatments, D1 and D2 irrigations were done with 180 ml and 120 ml water per pot daily, for 5 d. Then, the re-watered group following D2 treatment was supplied with normal water regime for 2 d and some plants were further cultured for another 5 d for biomass analysis. At each harvest, the plant was separated into shoots and roots. Shoots were cut at the cotyledon node, and fresh weight determined. Roots were measured by pulling pots from the ground and soaking the root mass in water, then manually stirring and pouring into a sieve (0.25 mm^2^ mesh). The sieve was suspended in a large water bath and shaken continuously until roots were washed free of soil. Soil materials remaining on the sieve were removed manually. The separated root fractions were collected to determine fresh weight. Then all samples were cured at 105°C for 30 min and dried at 70°C to determine the shoot and root dry weight. Fresh samples of all treatments were used for immediate assays or frozen in liquid nitrogen and stored at −80°C for physiological and biochemical analysis (see below).

### Water loss and stomatal aperture measurements

T4 transgenic and WT maize were grown in pots under well-watered conditions for 21 d and then drought stress was imposed for 5 d as described above. Leaves of maize were cut and transpirational water loss was measured as described by Chen et al. [47]. The uppermost fully expanded leaves of WT and T4 transgenic maize under drought treatments were used in the experiments. Stomatal bioassay was performed as described by Pei et al. [48] with slight modifications. Leaves were carefully cut into 10-mm long and 5-mm wide strips, and the strips were immediately incubated in FAA fixative liquid (38% formaldehyde, acetic acid and 50% alcohol, 5∶5∶90). Stomata were observed under a scanning electron microscope (S-570; Hitachi, Japan), and the width and length of stomatal apertures were measured using image analysis computer software (Scion Image; Scion Corp., Frederick, MD; and National lnstitutes of Health, Bethesda, MD). For each independent measurement, five stomata were selected to measure stomatal aperture**s** on each randomly selected digitized image from six sections of the abaxial surface.

### Leaf water potential, RWC, and ABA content

Leaf water potential of the uppermost fully expanded leaves of WT and T4 transgenic maize was taken on 0 d (21-d-old seedlings) and 5 d after initiation of different drought treatments and 2 d after re-watering as described above. A pressure chamber (Model 3000, Soil Moisture Equipment Corp., Santa Barbara, CA, USA) was used to measure the leaf water potential, with one leaf per plant and six plants per treatment. The RWC was measured as described by Gaxiola et al. [49]. Endogenous ABA content was measured by an indirect enzyme-linked immunosorbent assay (ELISA) as described by Yang et al. [50].

### Electrolyte leakage and MDA, proline and H_2_O_2_ content

The uppermost fully expanded leaves of WT and T4 transgenic maize, from 0 d (21-d-old seedlings) and 5 d after initiation of different drought treatments and 2 d after re-watering, were washed briefly in deionized water and 5-mm-diam leaf discs were punched out. Membrane damage was assayed by measuring ion leakage from leaf discs as described by Shou et al. [51]. The extent of lipid peroxidation was estimated by measuring the amount of MDA as described by Quan et al. [52]. Proline content was measured according to Bates et al. [53], and H_2_O_2_ content was measured as described by Brennan and Frenkel [54].

### SOD, POD, CAT, and AO enzyme assays

Fresh leaf segments from T4 transgenic and WT maize with different drought treatment were crushed into fine powder in a mortar and pestle under liquid nitrogen. Soluble protein content was determined following the Bradford method [55] with BSA as standard. Total SOD activity was assayed according to Giannopolitis and Ries [56]. POD activity was determined by the guaiacol oxidation method of Aebi [57]. CAT activity was measured following Nakano and Asada [58]. AO activity was measured by Native PAGE as described by Porch et al. [35]. Plant tissue was ground to a powder with liquid nitrogen and homogenized in ice-cold extraction buffer (250 mM TRIS-HCl, pH 7.5, 1 mM EDTA, 10 mM GSH, and 2 mM DTT,10 uM FAD, 50 uM leupetin, 80 uM sodium molybdate). A ratio of 1 g leaf tissue to 5 ml buffer (1∶5 w/v) was used, and homogenized plant material was centrifuged at 18 000 g and 4°C for 25 min. The resulting supernatant was subjected to native polyacrylamide gel electrophoresis (PAGE) on 7.5% polyacrylamide gels in a Laemmli buffer system in the absence of SDS at 4°C. Each lane in the gel was loaded with above 400 ug protein. After electrophoresis, the gel was immersed in 0.2 M phosphate buffer (pH 7.5) for 10 min, then AO activity staining was developed at room temperature in a mixture containing 0.1 M TRIS-HCl, pH 7.5, 0.1 mM phenazine methosulphate, 0.5 mM MTT (3 [4, 5-dimethylthiazol-2-yl] 2, 5-diphenyltetrazolium -bromide), and 1 mM substrate (1-naphthaldehyde or indole-3-aldehyde) in the dark for about 1 h. After activity attaining, the gels were scanned to quantify the relative intensity of formazan bands which were directly proportional to enzyme activity [59] using the Quantity One computer software in Bio-Rad ChemiDoc SRS (Bio-Rad, Hercules, CA, USA). Native PAGE was carried out with a Protein II xi Cell (JunYi, Beijing, China).

### Statistical analysis

Results are based on two independent experiments with at least three replicate tissue samples from three to four transgenic or WT plants in each treatment. Data were analysed using the Student's *t*-test (SPSS 13.0 for Windows; SPSS Inc., Chicago, IL, USA). Significant differences were determined based on *P*<0.05 or *P*<0.01.
